# Correlation of fasting and postprandial plasma glucose with HbA1c in assessing glycemic control; systematic review and meta-analysis

**DOI:** 10.1186/s13690-015-0088-6

**Published:** 2015-09-25

**Authors:** Ezra Belay Ketema, Kelemu Tilahun Kibret

**Affiliations:** Department of Biochemistry, College of Health Science, Mekelle University, Mekelle, Ethiopia; Department of Public Health, College of Medical and Health Science, Wollega University, Nekemte, Ethiopia

**Keywords:** Diabetes mellitus, Glycemic control, HbA1c, Fasting plasma glucose, Postprandial plasma glucose, Correlation

## Abstract

**Background:**

Glycemic control in diabetes mellitus is a cornerstone in reducing morbidity and mortality of the disease. Achieving glycemic control or reducing hyperglycemia significantly decreases the microvascular and macrovascular complications of diabetes. Even though measurement of glycated hemoglobin (HbA1c) remains the gold standard for assessment of glycemic control, there is no consensus whether fasting or postprandial plasma glucose (PPG) is a better predictor of glycemic control in resource-poor settings when HbA1c is not available. The aim of this systematic review and meta-analysis was to summarize evidences on the significance of fasting and postprandial plasma glucose, and their correlation with HbA1c.

**Methods:**

Relevant studies were identified through systematic search of online databases (e.g. EMBASE, MEDLINE/PubMed and Cochrane library) and manual search of bibliographies of the included studies. Original research papers describing the correlations or associations of fasting and postprandial plasma glucose with HbA1c were included. The MedCalc software was used for data entry and analysis. We used the random effect model to estimate the pooled correlations of fasting and postprandial plasma glucose with HbA1c. Heterogeneity assessment and robustness analysis was also performed.

**Result:**

From total 126 articles identified, 14 articles were eligible for systemic review. Eleven of these eligible studies evaluated the correlations of fasting and postprandial plasma glucose to the standard HbA1c values and used in meta-analysis. Seven of these studies (63.5 %) found better or stronger correlations between PPG and HbA1c than fasting plasma glucose (FPG). In all the studies that estimated the relative contribution FPG and PPG to the overall hyperglycemia, decreases in PPG was accounted for greater decrease in HbA1c compared with decreases in FPG value. PPG also showed a better sensitivity, specificity and positive predictive value than FPG. The pooled correlation coefficient (r) between PPG and HbA1c was 0.68 (*P* < 0.001, 95 % CI; 0.56–0.75) slightly higher than pooled correlation coefficient of FPG (*r* = 0.61(*P* < 0.001, 95 % CI; 0.48–0.72)).

**Conclusion:**

PPG has a closer association with HbA1c than FPG. Hence, PPG is better in predicting overall glycemic control in the absence of HbA1c.

**Electronic supplementary material:**

The online version of this article (doi:10.1186/s13690-015-0088-6) contains supplementary material, which is available to authorized users.

## Introduction

Glycemic control is the most important aspect in management of diabetes mellitus. It is a cornerstone in reducing morbidity and mortality of the diseases [1, 2]. The chronic hyperglycemia of diabetes is associated with long term damage, dysfunction, and failure of various organs, especially the eyes, kidneys, nerves, heart, and blood vessels [3]. Many large randomized clinical trials and observational studies in type 1 and 2 diabetes have clearly shown that achieving glycemic control or reducing hyperglycemia significantly decrease the microvascular and macrovascular complications of diabetes mellitus (DM) [4–6].

Control of plasma glucose in patients with diabetes can be assessed by measurement of glycated hemoglobin (HbA1c), fasting plasma glucose (FPG), and postprandial plasma glucose (PPG). However, still measurement of HbA1c level remains the gold standard for assessment of glycemic control at follow up [7]. The concentration of HbA1c predicts diabetes complications because it reflects more harmful glycation sequelae of diabetes, such as retinopathy and nephropathy, which are understood to be due to harmful advanced glycation end products [8–10]. Epidemiological and large randomized clinical trial studies such as Diabetes Control and Complication Trial (DCCT) and the United Kingdom Prospective Diabetes Study (UKPDS) indicated that HbA1c >7.0 % is associated with a significantly increased risk of both microvascular and macrovascular complications, regardless of underlying treatment [4, 5, 11]. Since it reflects the mean glycemic values in the previous 2–3 months, HbA1c is an indicator for overall glucose exposure integrating both fasting and postprandial hyperglycemia even though their relative contribution is undefined [12, 13]. Nevertheless, HbA1c test is not available or very limited in resource poor settings due to its high cost. In the absence of HbA1c test and high level of diabetic problems, post-prandial and fasting plasma glucose estimation have come into practice particularly in developing countries to assess glycemic control [6]. A number of studies have shown acceptable correlation between HbA1c levels and FPG and PPG level [14]. However, circumstantial evidence indicates that there is no consensus amongst professionals whether FPG or PPG is a better predictor of glycemic control in resource poor settings when HbA1c is not available [9, 15]. Nevertheless, for patients and health care providers, a clear understanding of the relationship between different plasma glucose measurements and HbA1c is necessary for setting appropriate day-to-day plasma glucose testing goals with the expectation of achieving specific HbA1c targets.

The aim of this systematic review and meta-analysis was therefore to summarize evidences on the significance of fasting and postprandial plasma glucose, and their correlation with HbA1c. This will help to identify the better surrogate glycemic marker for achieving target HbA1c level and for early detection of glycemic control status.

## Methods

### Data source and search strategy

We followed standard guidelines for the systematic review of diagnostic studies [16] and used the Preferred Reporting Items for Systematic Reviews and Meta-Analyses statement (Additional file 1: PRISMA) [17] as the template for reporting the review. Using guidelines and check lists of the standards we systematically identified and appraised peer-reviewed original research reports on comparison of correlations between fasting and 2-h postprandial plasma glucose with HbA1c in assessment overall glycemic control. A literature search was done for published articles using the online databases of EMBASE (www.embase.com), PubMed (www.pubmed.gov), Cochrane library (www.cochranelibrary.com/center), and google scholars (scholar.google.com) from their inception to February 2015. Bibliographies of included studies and international and national guidelines on diabetes mellitus management and diagnosis as well as unpublished articles were hand-searched for additional relevant studies. The databases were searched in January and February 2015.

The main search terms used individually or in combinations were: diabetes, hyperglycemia, type 1 diabetes or type 2 diabetes, hyperglycemia; fasting, pre-prandial hyperglycemia, basal hyperglycemia, Postprandial hyperglycemia, 2 h postprandial hyperglycemia, HbA1c or glycated hemoglobin, Glycemic control, glucose monitoring, diabetic control, glycemic target.

### Study selection

We reviewed and examined relevant articles that compared the correlation of fasting and 2- h postprandial plasma glucose with HbA1c value for eligibility beginning with titles and abstracts then followed by full text review.

### Eligibility criteria

The following inclusion criteria were applied: original studies; articles published in English; reported response rate ≥80 %; studies that compared the correlation of fasting and 2–hour Postprandial plasma glucose values to the standard HbA1c value, studies done in diabetic patients; studies that reported quality assurance methods.

Studies were excluded if compared fasting and 2–hour Postprandial plasma glucose values with mean glucose value, studies done in pregnant women; Primary studies that focused on effect of various treatment on fasting or postprandial plasma glucose or that aimed to determine the complication of fasting or postprandial plasma glucose were excluded from both systematic review and meta-analysis. Studies which did not report correlation coefficient (r) were excluded from Meta-analysis. The two investigators (EBK, KTK) assessed eligibility of all articles meeting the inclusion criteria through full text review. There was no any disagreement between the authors for the eligibility of selected articles.

### Data extraction /abstraction

Data extraction was performed by two independent researchers (EBK, KTK) using pretested and standardized abstraction form. Data were pulled out from each study for the following variables: title; first author; year of publication; study design; type of diabetes; cutoff points for fasting, postprandial plasma glucose and HbA1C; correlation coefficient (r) of fasting and 2 h postprandial plasma glucose with HbA1c; duration of diabetes; mean age of patients, mean HbA1c value; treatment set up, nutritional and other interventions; Methods of glucose and HbA1C analysis were also considered. When there was a disagreement in data abstraction, it was resolved through consensus between the two investigators.

### Definitions

Fasting and 2 h postprandial plasma glucose were defined as value of plasma glucose measured after overnight (8–12 h) fasting and after 2 h of meals respectively [1]. Cut off point values were considered as value which are used to classify the result of given parameter as normal (good) and abnormal (poor) [18]. HbA1c is a measure of the degree to which hemoglobin is glycosylated in erythrocytes and is expressed as a percentage of total hemoglobin concentration [9].

### Methodological quality assessment

Clearly described methods of patient selection and characteristics of the included patients, use of predefined good glycemic control cut off points for HbA1c, FBS and PPBS, description on how the index tests (FPG, and 2hPPG) and the reference standard (HbA1c) were conducted and interpreted, clear data collection methods and procedures, reported quality assurance methods (training of data collectors, pretesting, supervision, calibration, standardization of meals) and reporting of lost follow-up were considered as a study quality indicators.

### Heterogeneity assessment

The presence of statistical heterogeneity was evaluated using Cochran’s Q test (*P* < 0.10 reflected indicative of statistically significant heterogeneity) and magnitude of statistical heterogeneity between studies was assessed using I^2^ (values of 25 %, 50 % and 75 % were considered to represent low, medium and high heterogeneity respectively) [19].

### Data synthesis and statistical analysis

The descriptions of original studies were summarized using Tables and forest plot. The MedCalc (1993–2015) software was used for data entry and analysis. The random effect model was used to estimate the pooled correlation considering any heterogeneity inherent in the meta-analysis. The estimated pooled correlation was presented with the 95 % confidence interval (CI). We also performed robustness analysis to assess the impact each study on the pooled result by removing one at a time from the analysis for all studies.

## Result

### Identified studies

**Fig. 1:**
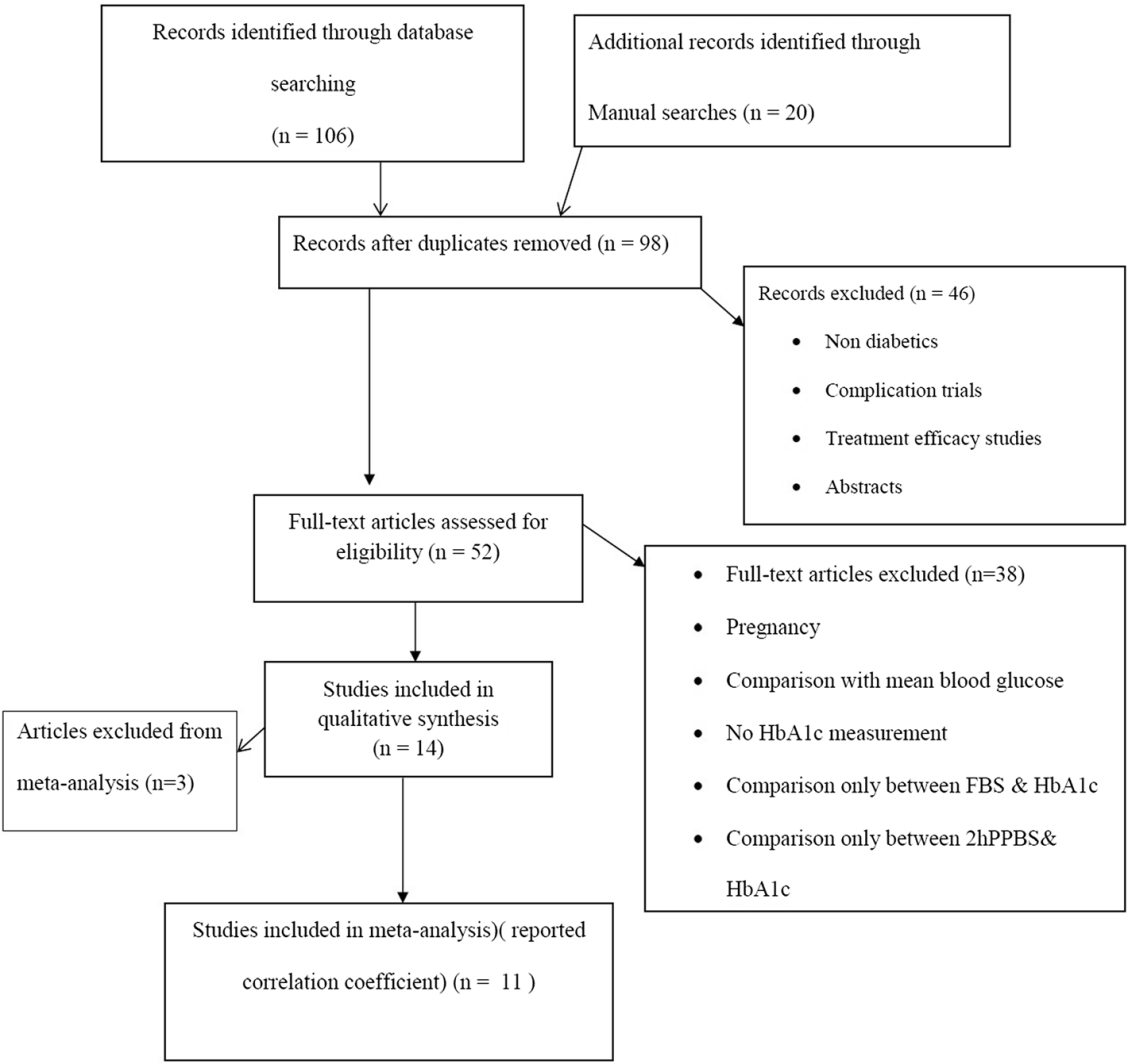
Flow diagram of search strategies

We identified 126 articles by the electronic search in MEDLINE/PubMed, Google scholar, Cochrane central Library and reference lists of included studies. Of the total, 112 were excluded after reviewing titles, abstracts and full text and applying the inclusion criteria. Fourteen studies were included in systematic review. From these 14 articles, only 11 of them were incorporated in meta analysis (Fig. 1). All of the included articles had performed comparisons between fasting and postprandial blood 166 glucose with HbA1c.

### Characteristics of included studies

Publication dates ranged from 1997–2014 with more publications done in 2014 (*n* = 3). Data from a total of 4007 and 2403 diabetic patients were included in this systematic review and meta-analysis, respectively. The included articles had study population varied from 50 (Gupta et al. 2014) to 1186 (Swetha et al., 2014) and were conducted from 9 different countries. Mean age of the study subjects ranged from 48.4 (Saiedullah et al., 2013) to 62.4 years (Woerle et al., 2007) although most were in between 55 and 60 (*n* = 7). As shown in Table 1 below almost all of the included studies (*n* = 13) used a cross-sectional study design. While eleven studies were done in type 2 diabetic patients exclusively, the rest 3 studies included both type 1 and type 2 diabetic patients.

**Table. 1:** Characteristics of studies included in systematic review and meta-analysis

Studies	Citation	Type of diabetes	Female proportion (%)	Mean age or range	Treatment modality	Sample size	Study design	Study site
Saeed, 2006	[20]	Type 2^a^	56	55.66	Oral antidiabetic	97	cross sectional	Sudan
Haddadihneshad et al., 2010	[21]	type 1 & 2	57.7	12-67 (range)	insulin & oral antidiabetic	300	Cross sectional	Iran
Rosediani et al., 2006	[13]	Type 2	53.7	56.7	Oral antidiabetic	82	cross sectional^b^	Malaysia
Swetha et al., 2014	[6]	Type 2	38.6	55.43	NS	1186	cross sectional	India
Gupta et al., 2014	[22]	Type 2	NS	NS	Oral antidiabetic	50	Cross sectional	Italy
Datta et al.,2014	[23]	type 1 & 2	53.3	51	Oral antidiabetic	120	cross sectional	India
Avignon et al., 1997	[24]	Type 2	36.3	40 - 78 (range)	Oral antidiabetic	66	cross sectional^b^	France
Bonora et al., 2001	[25]	Type 2^a^	43.1	59.5	Oral antidiabetic	371	cross sectional^b^	Italy
Shrestha et al., 2012	[26]	Type 2	53.3	58.9	Oral antidiabetic	60	cross sectional^b^	India
Azim et al., 2011	[27]	type 1 & 2	35.0	51.8	NS	71	cross sectional	Pakistan
Saiedullah et al., 2013	[28]	Type 2	44.1	48.4	Oral antidiabetic	177	cross section^b^	Bangladesh
Woerle et al., 2007	[29]	Type 2	45.1	62.4	Insulin & antidiabetic	164	Prospective; intervention	Germany
Schernthaner et al., 2010	[30]	Type 2	46.4	56.7	Oral antidiabetic	973	cross sectional	Multi center
Monnier et al.,2003	[12]	Type 2^a^	52.0	60.1	Oral antidiabetic	290	cross sectional	France

^a^:constant diets and/or drugs were used for at least 3 months before the study; ^b^:controlled or standardized meal before testing; *NS* not specified

Some of the studies (Schernthaner et al., 2010; Saeed, 2006; Monnier et al.,2003; Bonora et al., 2001) included only patients who had not changed their treatment and dietary habits while Schernthaner et al., (2010) and Woerle et al., (2007) used unsatisfactory glycemic control (HbA1c > 7.5 % & 6.5 %, respectively) as their main inclusion criteria. Majority of studies (*n* = 9) were done on diabetic patients taking non-insulin oral anti diabetic treatment. Only five of the included studies stated interventions they undertaken (4 food or diet and 1 intensive treatment) while the rest (*n* = 9) did not specify or carried out any interventions (Table 1).

### Plasma glucose and HbA1c measurements

In 86 % of the included studies plasma glucose was determined by glucose oxidase-peroxidase enzymatic method in automated clinical chemistry analyzers. Two studies, Woerle et al. (2007) and Schernthaner et al. (2010), used glucose data measured by self-monitoring glucose device. HbA1c was measured using ion exchange high-performance liquid chromatographic techniques in all included studies except two studies, Gupta et al. (2014) and Datta et al., (2014), who used immunoturbidimetric methods. Six of the included studies used two blood samples each for FPG and 2hPPG determination taken before and after 2 h of breakfast, respectively. The other five studies employed multiple plasma glucose measurements per day before and after each meal (breakfast, lunch and dinner) while Saeed (2006) and Haddadihneshad et al. (2010) used the average value of 3 separate plasma glucose measurements done on different days. But Shrestha et al. (2012) used the average of plasma glucose measured for 15 consecutive days (Table 2).

**Table. 2:** Sensitivity, specificity and positive predictive value (PPV) of fasting and postprandial plasma glucose tests in four studies included in systematic review

Studies	Citation	Sensitivity (%)		Specificity (%)		PPV (%)	
		FPG	PPG	FPG	PPG	FPG	PPG
Rosediani et al., 2006	[13]	81	75	58.3	80.6	70.6	82.5
Swetha et al., 2014	[6]	74	79	84	74	87	80
Datta et al., 2014	[23]	85	92	81	90	89	95
Avignon et al., 1997	[24]	69	73	85	92	62	76
Overall result		PPG is more sensitive		PPG is more specific		PPG has higher predictive value	

*FPG* fasting plasma glucose, *PPG* Postprandial plasma glucose, *PPV* Positive predictive value

### Outcome measures and summary of findings

Most of the included studies (*n* = 11) calculated Pearson’s correlation coefficient to measure the strength of association between FPG or PPG and HbA1c. From these, seven studies found a better correlation between PPG and HbA1c than FPG. In contrast to this, the other three studies revealed a stronger correlation between FPG and HbA1c than PPG. The remaining one study found almost equal correlation coefficients for both tests. The correlation coefficient (r) ranged from 0.20–0.86 for PPG and from 0.28–0.84 for FPG. Both the maximum(*r* = 0.86) and minimum (*r* = 0.20) values was found in PPG. All studies reported a statistically significant (*p*- value < 005) correlation between PPG or FPG and HbA1c.

Alternatively, Woerle et al. (2007), Schernthaner et al. (2010), Monnier et al.,(2003) employed a different approach to estimate the relative contribution of FPG & PPG to the overall glycemia. Woerle and his colleagues found that only 64 % patients achieving FPG targets of <100 mg/dl achieved an HbA1c target of <7 % whereas 94 % of patients achieving the postprandial target of <140 mg/dl did. A decrease in PPG was accounted for nearly twice as much as FPG did for the decreases in HbA1c. On the other hand Schernthaner et al. (2010) described the dependency of relative contribution of FPG & PPG to the overall glycemia by HbA1c values. The authors stated that PPG play a major role in patients suffering from mild or moderate hyperglycemia while FPG appears as a main contributor to the overall diurnal hyperglycemia in poorly controlled diabetic patients. Similar suggestions were also made by Monnier and his co-authors. Collectively, the data from these three studies indicated that PPG contributes more than FPG to overall hyperglycemia as control of PPG was found essential either to decrease or to obtain HbA1c goals of <7.

Only four studies calculated the specificity, sensitivity and positive predictive value of FPG and 2hPPG tests to detect the change in HbA1c values in addition to Pearson’s correlation. Of these four studies, three of them found better sensitivity, specificity and positive predictive value for PPG than FPG (Table 2). This indicates that PPG is more sensitive, more specific and has a higher predictive value than FPG. However, these studies used a different cut of value for both FPG and PPG. Datta et al., (2014) also determined the measure of accuracy of the two tests and found higher value for PPG (92 %) than FPG (83 %) (Table 2).

Generally in all of the three approaches or methods, PPG was found to be strongly correlated or to be a better parameter in predicting or achieving target HbA1c values than FPG. Summary of the results showed that PPG has a better correlation with HbA1c, superior accuracy (sensitivity and specificity) to predict HbA1c values and greater contributions to achieve target HbA1c values than FPG.

### Meta analysis

**Fig. 2:**
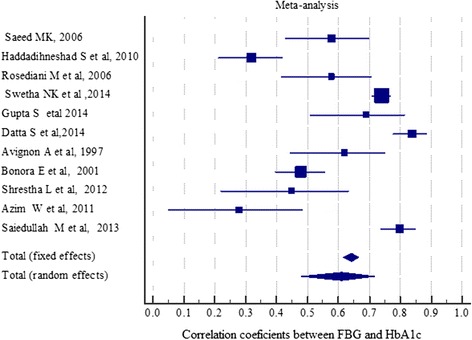
Forest plot of the 11 studies that quantitatively assessed the correlation between HbA1C and FPG represented by the random effect model. Size of the square is proportional to the precision of the study-specific effect estimates, and the bars indicate the corresponding 95 % CIs. The diamond is placed on the summary correlation coefficient of the observational studies, and the width indicates the corresponding 95 % CI

**Fig. 3:**
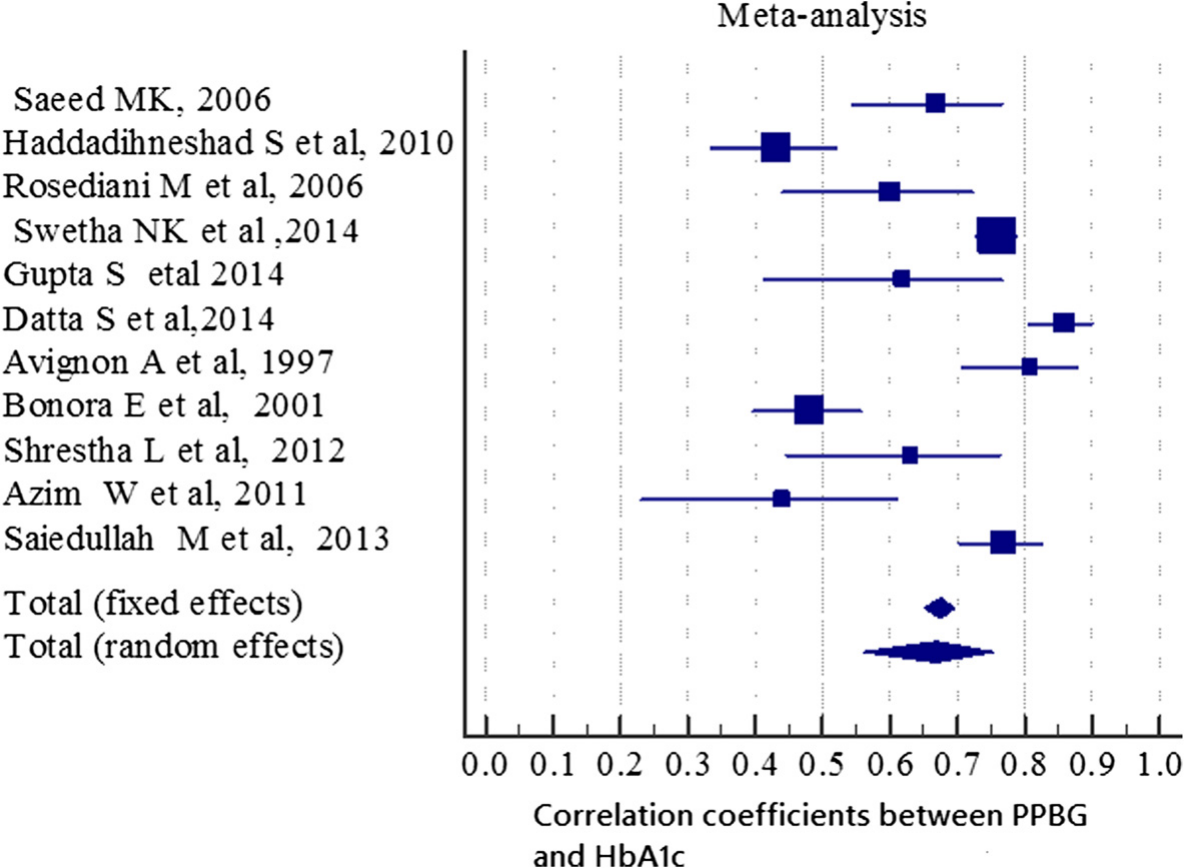
Forest plot of the 11 studies that quantitatively assessed the correlation between HbA1C and PPG represented by the random effect model. Size of the square is proportional to the precision of the study-specific effect estimates, and the bars indicate the corresponding 95 % CIs. The diamond is placed on the summary correlation coefficient of the observational studies, and the width indicates the corresponding 95 % CI

Meta-analysis was done on eleven selected studies listed in Table 3. All the studies included in meta-analysis quantitatively measured the relationship between FPG and 2hPPG and the standard HbA1c values using Pearson’s correlation coefficient (r value). Seven of the studies included in meta-analysis found better correlations between 2hPPG and HbA1c while the remaining 3 studies reported better correlation between FPG and target HbA1c values as shown in Table 3. Based on meta-analysis of these 11 included studies the pooled correlation coefficient was 0.61(95 % CI; 0.48–0.72) for FPG and 0.68 (95 % CI; 0.56–0.75) for 2hPPG using random effect model as presented in Figs. 2 and 3. Forest plot for correlations of FPG and 2hPPG with HbA1c is shown in Figs. 2 and 3, respectively. Forest plot for correlations of FPG and 2hPPG with HbA1c is shown in Figs. 2 and 3, respectively. Size of the square is proportional to the precision of the study-specific effect estimates, and the bars indicate the corresponding 95 % CIs. The diamond is placed on the summary correlation coefficient of the observational studies, and the width indicates the corresponding 95 % CI

Since the included studies used a different cut off points for FPG and PPG, we didn’t perform meta analysis for sensitivity, specificity and positive predictive value of FPG and PPG (Table 3). The use of different cut off points for defining good glycemic control affects the sensitivity, specificity and positive predictive values of FPG and PPG tests.

**Table. 3:** Characteristics of Studies included in meta-analysis

Studies	Type of DM	Study design	Cut off points for good glycemic control (mg/dL)			Correlation between HbA1c and	
			FPG	PPG	HbA1c (%)	FPG	PPG
Saeed, 2006	Type 2	cross sectional	120	160	7 %	0.60	0.20
Haddadihneshad et al., 2010	Type 1 & 2	cross sectional	120	160	ND	0.32	0.43
Rosediani et al., 2006	Type 2	cross sectional	110	145	7 %	0.58	0.60
Swetha et al., 2014	Type 2	cross sectional	130	180	7 %	0.74	0.76
Gupta et al., 2014	Type 2	cross sectional	120	140	6.5 %	0.68	0.62
Datta et al., 2014	Type 1 & 2	cross sectional	110	126	7 %	0.84	0.86
Avignon et al., 1997	Type 2	cross sectional	120	140	≤7.0 %	0.62	0.81
Bonora et al., 2001	Type 2	cross sectional	120	160	7 %	0.48	0.48
Shrestha et al., 2012	Type 2	cross sectional	120	200	>6.5 %	0.45	0.63
Azim et al., 2011	Type 1 & 2	cross sectional	126	200	>6.5 %	0.28	0.44
Saiedullah et al., 2013	Type 2	cross section	ND	ND	ND	0.81	0.77

*DM* diabetes mellitus, *ND* cut off value not defined, *HbA1c* glycated hemoglobin, *FBS* fasting plasma glucose, *PPBS* postprandial plasma glucose

### Heterogeneity assessment

The 11 studies included in meta-analysis showed high heterogeneity according to Cochrane Q and I^2^ test statistic. Q test, *p* < 0.001 and I^2^ test = 94.3 % was found for FPG. Similarly Q test *p* < 0.001 and I^2^ test = 93.2 % was for PPBS which are indicatives for using random effects model. We also performed robustness analysis by revolving one article at a time. But each study did not bring significance difference on the final pooled correlation coefficient.

## Discussion

Historically, glycemic control efforts have emphasized on achievement of HbA1c and FPG targets [31]. Unfortunately majority (more than two thirds) of patients in therapeutic aims targeting for HbA1c and FPG has failed to achieve their glycemic goals [1, 32]. On the other hand, HbA1c has some important limitations and is a rather complex measure of hyperglycemia. A large number of medical condition such as the presence of hemoglobin variants, malignancies, hemolytic anemia, and variety of systemic conditions as well as various medications and pregnancy are associated with alterations in the HbA1c values and may provide unreliable information [33, 34]. Apart from these factors, HbA1c also neither captures glucose fluctuations over short period of time nor provide any information on glucose dynamics [18]. But these glycemic variability are critical for safe and timely treatment adjustment and clinical decision makings [35]. As a result, there has been increasing interest in additional markers for better glycemic control over shorter timeframes [18].

In this review we identified 14 articles that equated the relationship between short term glucose measures, FPG and 2 h PPG, and long term glycemic indicator HbA1c. According to this review and meta-analysis a better correlation was found between 2 h PPG and HbA1c than FPG (pooled correlation (r) 0.67 Vs 0.61). This means that patients who achieved 2 h PPG within the reference limit will better accomplish target HbA1c values than patients realized FPG within recommended range.

Some of the included studies indicated that PPG levels made the highest contribution in the lower HbA1c quintile (in good or fair HbA1c values whereas fasting hyperglycemia appeared as the main contributor to the overall hyperglycemia in patients with poorly controlled disease (HbA1c > 9 %). In other expression it means that decreases in PPG accounted for greater decrease in HbA1c compared with decreases in FPG so that control of PPG is an important consideration for achieving recommended HbA1c goals <7 % than FPG. Three out of four studies also found a better sensitivity, specificity, and positive predictive value for PPG. This can be interpreted as when PPG is high or its control is poor it is more likely to get high HbA1c above the recommended range. Our review shad that in all the circumstances PPG is either a better correlate or accurately predicts HbA1c value or its contribution to the overall hyperglycemia is greater than FPG signifying that control of postprandial hyperglycemia is essential for achieving recommended HbA1c goals.

Growing body of evidences have also shown a strong association between PPG and cardiovascular risk and outcomes [36], oxidative stress, carotid intimal thickness and endothelial dysfunction [37]. A recent diabetes complications trial study concluded that PPG, but not FPG, was an independent predictor of mortality and cardiovascular complications in diabetes [36, 38, 39]. It is also plausible that humans spend half of their lives in postprandial states and thus, to achieve better long-term metabolic control (HbA1c) and minimize the risk of chronic diabetic complications, glucose monitoring in postprandial state will be indispensable.

However, this review was also based on most data collected from type 2 (*n* = 11) and few (*n* = 3) from both type 1 and type 2 diabetic patients. Considering the biochemical and pathological differences in the two types, a distinct approach would be appropriate to characterize these associations in type 1 diabetic patients. Regarding day to day variations numerous evidences suggested that several determination of glucose over a period of several weeks would be better indicator of glycemia and then better correlated with HbA1c than single or few measurements in single day. But in this review only three studies (Saeed, 2006; Haddadihneshad et al., 2010 and Shrestha et al., 2012) used the average value of 3 or more separate plasma glucose measurements done on different days. All others were based on either glucose data generated on single day or single glucose measurement. None of the included studies identified the separate effect of age and sex on the association between these different plasma glucose profiles and HbA1c values. However Szoke et al. found that older people have more postprandial hyperglycemia than younger people [40]. Finally significant heterogeneity was found in the pooled correlation estimates of both FPG and PPG. Even though we attempted to explain it through the random effect regression model, many factors possibly contributing to this residual heterogeneity could not be assessed because they were not reported in most studies. For example, duration of diabetes and type of treatments are likely to have an important effect on correlation results. Finally it should not be also ignored that methodological differences may contribute to the study to study variation. It is suggested that the multiple regression analysis used for studying the relationship between A1C and glucose values at different times is an unstable model when explanatory variables, i.e., the glucose values in this case, are inter correlated [41].

### Limitation

Inclusion of different studies from different geographical location, treatment group as well as from different methodological approach would be problematic to end up with particular inferences. Lack of uniform cut off points for good glycemic control across the studies disallowed us to pool the specificity and sensitivity of FPG and PPG in predicting HbA1c values. And also due to incomplete data on inclusion criteria, duration of diabetes, age groups and others it was difficult to perform subgroup analysis. Another constraint in this meta analysis could arise from publication bias as studies that have negative result are less likely to be published.

## Conclusion

The result of our reviews showed that PPG strongly correlate with HbA1c or contributes significantly to overall glycemic control. This is in line with contemporary evidence that showed strong relation between PPG and development of diabetes complications. Consequently we in a position to claim that special attention should be given to monitoring and treating PPG until the ongoing debate are resolved through large randomized control trials. Hence monitoring of PPG will be more helpful to achieve optimal glycemic control and prevent long term diabetes complication than FPG alone in the absence of HbA1c, especially in developing countries.

## Additional file

Additional file 1:
**Annex I.** PRISMA 2009 Checklist. (PDF 102 kb).

## Abbreviations

HbA1c: Glycated Hemoglobin
FPG: Fasting Plasma glucose
PPG: Postprandial Plasma glucose
PPV: Positive Predictive Value
2hPPG: 2-h Postprandial Plasma glucose
